# Case Report: Copper sulphate related hemophagocytosis with lymphohistiocytosis

**DOI:** 10.12688/f1000research.147189.1

**Published:** 2024-04-23

**Authors:** Koushik Ramachandra, Amruth Reddy Narayana, Samyuktha Srinivas, Sridevi H B

**Affiliations:** 1 Department of General Medicine, Kasturba Medical College, Mangalore, Manipal Academy of Higher Education, Manipal, India; 2 Department of Pathology, Kasturba Medical College, Mangalore, Manipal Academy of Higher Education, Manipal, India

**Keywords:** Hemophagocytic Lymphohistiocytosis (HLH), Copper Sulphate, Immune-mediated, Intravascular hemolysis, Acute Kidney Injury

## Abstract

The accidental, suicidal, and homicidal toxicities of copper sulfate have been extensively documented. The later stages of the disease demonstrate signs of systemic toxicity, characterized by intravascular hemolysis, oliguric renal failure, convulsions, and circulatory collapse. Despite the extensive description of life-threatening intravascular hemolysis, Hemophagocytic Lymphohistiocytosis (HLH) related to copper sulfate poisoning has not been described.

A 45-year-old male presented with accidental consumption of copper sulfate. Laboratory evaluation revealed leukocytosis, intravascular hemolysis, acute liver injury, acute kidney injury, severe metabolic acidosis, and hyperkalemia. The patient was shifted to the Intensive Care Unit and hemodialysis was initiated. On the 9 ^th^ day, he developed high-grade fever with chills. With the suspicion of a central line-associated bloodstream infection, empirical antibiotic therapy was initiated, and the lines were revised. On the 19 ^th^ day, the high-grade fever recurred. Investigations revealed trilineage cytopenias. With a high degree of suspicion for HLH, further investigations revealed increased ferritin levels. Bone marrow aspiration cytology showed evidence of reactive marrow with haemophagocytic lymphohistiocytosis. The patient was initiated on corticosteroid therapy, after which symptomatic and laboratory recovery was noted.

Although copper sulfate poisoning is potentially fatal in large quantities, few studies have examined the possible immune-mediated abnormalities in individuals. Owing to the direct membranolytic effect of copper sulfate, it is not unreasonable to have immune-mediated organ damage. To the best of our knowledge, this is the first report of Hemophagocytic Lymphohistiocytosis attributed to copper sulfate intoxication.

The present case demonstrates that the diagnosis of HLH must be considered when treating a case of copper sulfate poisoning; however, the exclusion of the most common complications must be first established.

## Introduction

Acute copper intoxication is often the result of accidental consumption, suicide, or homicidal attempts. Bordeaux mixture, a copper-based fungicide used in agricultural practice, is widely available in the Indian sub-continent. ^1^^,^^2^ Ingestion of more than 1 g of copper sulfate results in manifestations of symptoms while–20 g may be lethal. ^3^^,^^4^ The clinical manifestations of toxicity predominantly involve erosive gastropathy, hepatitis, acute kidney injury, rhabdomyolysis, convulsions, circulatory collapse, and haematological complications. ^5^

The major haematological manifestations include intravascular Haemolysis and Methemoglobulinemia. ^5^ Intravascular hemolysis often starts as early as 12-24 hours after ingestion of the substance owing to the direct membranotoxic and oxidative stress caused to the erythrocyte membrane, whereas methemoglobinemia occurs because Cu ^2+^ ions oxidize Fe ^2+^ in hemoglobin to Fe ^3+^. ^6^ Although these two manifestations have been described extensively in the literature, the occurrence of Hemophagocytic Lymphohistiocytosis (HLH) due to copper sulfate ingestion has not been described.

HLH is a disease of multi-organ dysfunction that can have extremely nonspecific presentations. ^7^ These include prolonged fever, cytopenia, hepatosplenomegaly, liver dysfunction, and neurological dysfunction. ^8^ The broad range of presentations and lack of specificity make the diagnosis of HLH quite challenging, requiring the ruling out of alternative diagnoses. ^7^

Herein, we describe a case of Hemophagocytic Lymphohistiocytosis attributed to accidental copper sulfate poisoning.

## Case report

A male in his 40s with an unremarkable medical history was referred to our center with an accidental, alleged consumption of copper sulfate while under the influence of alcohol. He was treated at a nearby regional hospital with gastric lavage and intravenous fluids before referral. On reporting, the patient was conscious, well-oriented, and hemodynamically stable. Tachypnea, pallor, and icterus were also noted. Initial laboratory evaluation revealed anemia with a hemoglobin level of 12.6% and leukocytosis with a white blood cell count of 23500 cells/μL, evidence of hemolysis, acute liver injury, acute kidney injury, and severe metabolic acidosis with hyperkalemia. The patient was admitted to the Intensive Care Unit for further care.

The following day, his hemoglobin dropped to 6.9 gm%, the leukocyte count increased to 41400 cells/μL, and platelet count dropped to 1.01 lakh cells/μL. His serum creatinine elevated by 2.96 units and serum urea by 70 units above the baseline, suggesting worsening renal function owing to the extensive damage caused to the kidney by copper sulfate. Hyperkalemia persisted. The patient remained oliguric with increasing acidosis and was initiated on supportive hemodialysis. He underwent six cycles of hemodialysis during his hospital stay. Supportive blood transfusions were also started.

On the 9 ^th^ day of hospitalization, the patient developed high-grade fever with chills. With suspicion of Central Line Associated Blood Stream Infection (CLABSI), he was started on empirical antibiotics following collection of appropriate cultures, and the lines were revised. No growth was observed in the culture medium. The patient continued to be dialysis dependent due to ongoing oliguric renal failure.

**Figure 1.**
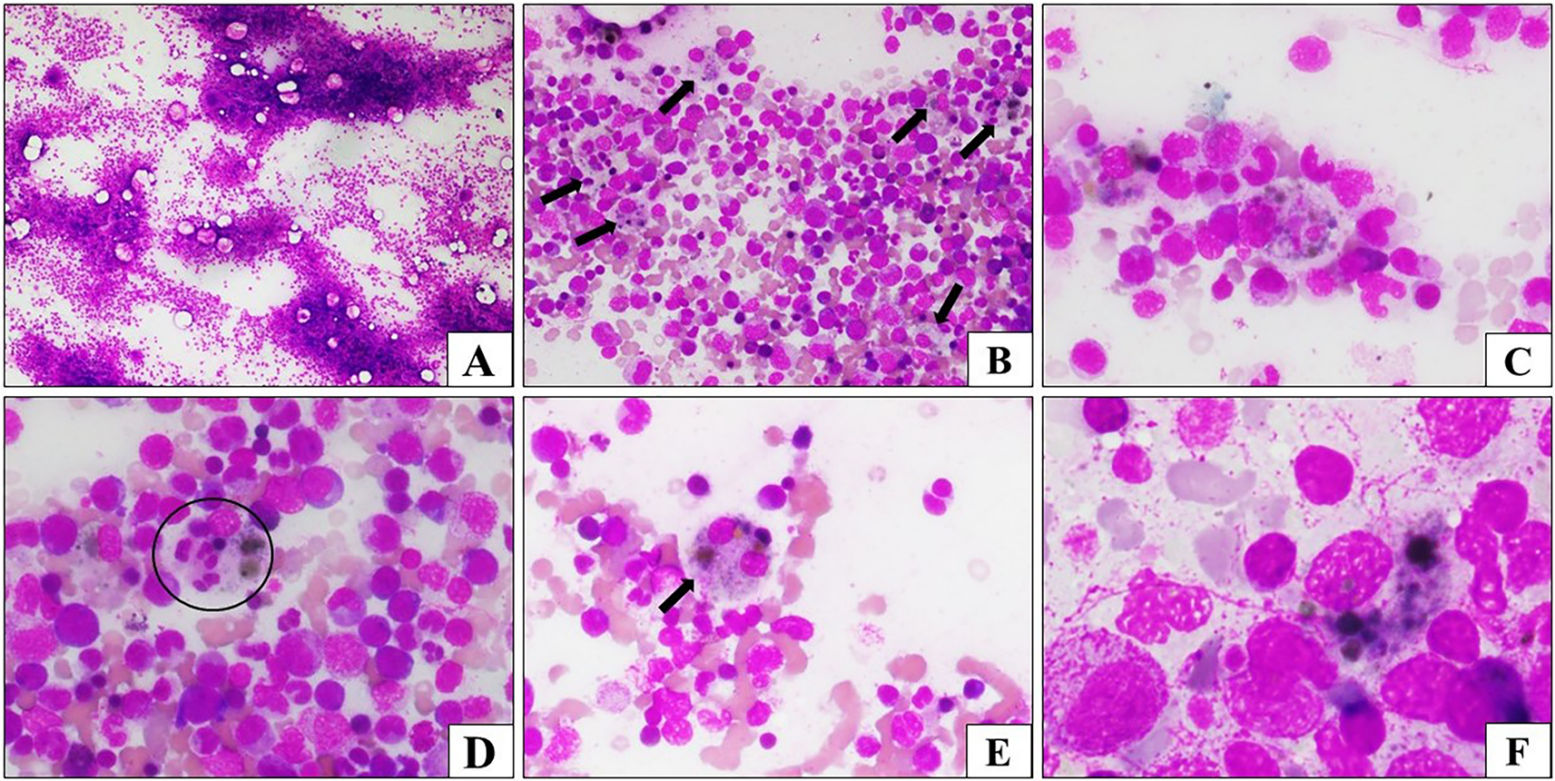
(A-F): Bone marrow aspirate: A: Highly cellular bone marrow particles (Leishman Stain; 40X). B: Increased marrow macrophages (arrow), many with pigment or engulfed cells (Leishman stain;100X). C: Hemophagocytic macrophages with evidence of cellular remnants within the cytoplasm (Leishman stain;400X). D: Active hemophagocytosis: Macrophage (encircled), has engulfed lymphocyte, erythroblast and neutrophils (Leishman stain;400X). E: Pigmented hemophagocyte (Leishman stain;400X). F: Increase in pigment-laden macrophages in the marrow (Leishman stain;1000X).

On the 19 ^th^ day of hospitalization, a similar episode of high-grade fever with normal leukocyte counts recurred, along with anemia. A peripheral Smear was unremarkable. Blood cultures were resent, and the lines were revised. However, the fever persisted. As a result of persistent fever, investigations revealed trilineage cytopenia, and HLH was suspected. Targeted investigations have revealed increased serum ferritin and D-dimer levels. The bone marrow aspirate showed evidence of reactive marrow with Hemophagocytic Lymphohistiocytosis, as depicted in Figure 1. Repeated blood cultures were considered sterile. The patient was initiated on corticosteroids for HLH, following which his fever subsided, with improvement in cytopenia. Supportive care was then continued. Renal function tests showed gradual recovery with improved urine output. The dialysis sessions were gradually spaced, stopped, and then discharged. He was followed up as an outpatient until his kidney function normalized.

Serial investigations are tabulated in Table 1.

**Table 1.** Summary of laboratory findings.

Date	Hemodialysis	Hemoglobin	Total leukocyte count	Platelet count	Serum creatinine	Blood urea	Serum potassium
		gm/dL	cells/μL	cells/μL	mg/dL	mg/dL	mEq/L
23.07	-	12.6	26300	2.29L	1.49	31	5.17
24.07	-	6.9	41400	1.01L	4.45	200	5.2
25.07	Yes	5.21	41800	0.79L	9.25	347	
27.07	Yes	3.4	28900	1.5L	7.73	238	4.47
29.07	Yes	6.9	30850	1.4L	6.84	212	
02.08	Yes	5	21980	2.34L	9.19	212	
07.08	-	4.8	4780	2.56L	13.5	187	5.53
08.08	Yes	6.5	3170	1.98L	9.15	115	3.81
10.08	-	5.9	5310	1.44L	12.6	137	
12.08	Yes	8.5	2910	1.4L			
18.08	-	9.6	18840	1.4L	9.68	42	
20.08	-	8.9	11630	1.08L	5.45	48	
24.08	-	7.7	4410	1.27L	5.72	77	
29.08	-				3.9	97	
10.09	-	10.2	8610	1.52L	1.6	42	

## Discussion

Copper sulfate, a herbicide commonly used in agricultural practice in the Indian subcontinent, is commonly abused in suicidal or homicidal attempts. ^9^ Following the initial symptoms of metallic taste, salivation, thirst, nausea, abdominal pain, recurrent vomiting, and painful diarrhea, signs of systemic toxicity appear, which are characterized by intravascular hemolysis, oliguric renal failure, convulsions, and circulatory collapse.

HLH, often described as a diagnostic conundrum, is a life-threatening manifestation of uninhibited activation of immune cells to secrete excessive amounts of cytokines, leading to tissue destruction and multi-organ failure. ^10^ HLH can be of two types: familial and sporadic. The latter tends to occur with autoimmunity, malignancies, infection. ^8^ Common clinical manifestations include prolonged fever, erosive gastropathy, hematemesis, melena, acute liver failure due to direct copper toxicity, hepatosplenomegaly, anuria following acute kidney injury, and neurologic dysfunction, such as seizures, ataxia, and paraplegia. ^5^ Elevated serum ferritin, pancytopenia, deranged liver function tests, kidney function tests, elevated D-dimer, hypertriglyceridemia, and hypofibrinogenemia are laboratory abnormalities that should be considered when diagnosing HLH. ^11^^–^^13^

Here, we describe the case of a male in his 40s with an alleged accidental consumption of an unquantified amount of copper sulfate who developed features of HLH during his hospital stay. Although the presence of intravascular hemolysis-related complications, which occur as early as the first 12-24 hours have been extensively documented in copper sulfate poisoning, the occurrence of HLH has not been reported. Few studies have described possible immune-mediated abnormalities in individuals with copper sulfate poisoning.

On arrival, the patient experienced intravascular hemolysis, leukocytosis, acute kidney injury, acute liver injury, and severe metabolic acidosis with hyperkalemia. He underwent supportive transfusions and six cycles of hemodialysis. There are multiple factors contributing to the development of AKI in copper sulfate toxicity, including direct oxidative damage to the proximal tubules, hemoglobinuria, rhabdomyolysis, and pre-renal failure. ^14^ Recurrent episodes of fever after repeated line revisions and culture negativity raised the suspicion of HLH. Bone marrow aspiration cytology and elevated D-dimer and ferritin levels confirmed the suspicion of HLH and specific treatment was initiated. The patient recovered with the normalization of renal function.

To the best of our knowledge, this is the first report of Hemophagocytic Lymphohistiocytosis attributed to copper sulphate toxicity. The present case demonstrates that HLH could be one of the differential diagnoses for fever in such patients, although it should be considered after the exclusion of common etiologies.

## Ethical approval

Ethical approval not required.

## Consent

Written informed consent was given by the patient.

## Data Availability

No data are associated with this article.

## References

[1] Agrios GN : *Plant pathology.* Amsterdam: Elsevier Academic;2004.

[2] Bagchi D Bagchi M : *Metal toxicology handbook.* Boca Raton: CRC Press, Taylor & Francis;2021.

[3] Sinkovic A Strdin A Svensek F : Severe acute copper sulphate poisoning: a case report. *Arh. Hig. Rada Toksikol.* 2008 Mar;59(1):31–35. 10.2478/10004-1254-59-2008-1847 18407869

[4] Ellenhorn MJ Barceloux DG : *Ellenhorn’s medical toxicology: Diagnosis and treatment of human poisoning.* Baltimore: Williams & Wilkins;1997.

[5] Saravu K Jose J Bhat MN : Acute ingestion of copper sulphate: A review on its clinical manifestations and management. *Indian J. Crit. Care Med.* 2007;11(2):74–80. 10.4103/0972-5229.33389

[6] Trottestam H Horne A Aricò M : Histiocyte Society. Chemoimmunotherapy for hemophagocytic lymphohistiocytosis: long-term results of the HLH-94 treatment protocol. *Blood.* 2011 Oct 27;118(17):4577–84. Epub 2011 Sep 6. 10.1182/blood-2011-06-356261 21900192 PMC3208276

[7] Kaçar AG Celkan TT : Hemophagocytic Lymphohistiocytosis. *Balkan Med. J.* 2022 Sep 9;39(5):309–317. 10.4274/balkanmedj.galenos.2022.2022-4-83 35965424 PMC9469671

[8] Gamakaranage CS Rodrigo C Weerasinghe S : Complications and management of acute copper sulphate poisoning; a case discussion. *J. Occup. Med. Toxicol.* 2011 Dec 19;6(1):34. 10.1186/1745-6673-6-34 22182712 PMC3269987

[9] Grzybowski B Vishwanath VA : Hemophagocytic Lymphohistiocytosis: A Diagnostic Conundrum. *J. Pediatr. Neurosci.* 2017 Jan-Mar;12(1):55–60. 10.4103/jpn.JPN_140_16 28553383 PMC5437791

[10] Niece JA Rogers ZR Ahmad N : Hemophagocytic lymphohistiocytosis in Texas: observations on ethnicity and race. *Pediatr. Blood Cancer.* 2010 Mar;54(3):424–428. 10.1002/pbc.22359 19953651

[11] Okamoto M Yamaguchi H Isobe Y : Analysis of triglyceride value in the diagnosis and treatment response of secondary hemophagocytic syndrome. *Intern. Med.* 2009;48(10):775–781. Epub 2009 May 15. 10.2169/internalmedicine.48.1677 19443971

[12] Fukaya S Yasuda S Hashimoto T : Clinical features of haemophagocytic syndrome in patients with systemic autoimmune diseases: analysis of 30 cases. *Rheumatology (Oxford).* 2008 Nov;47(11):1686–1691. 10.1093/rheumatology/ken342 18782855

[13] Dash SC : Copper sulphate poisoning and acute renal failure. *Int. J. Artif. Organs.* 1989 Oct;12(10):610. 10.1177/039139888901201002 2807586

[14] Malik M Mansur A : Copper sulphate poisoning and exchange transfusion. *Saudi J. Kidney Dis. Transpl.* 2011 Nov;22(6):1240–1242. 22089793

