# Supplementary material for: Case Report: Copper sulphate related hemophagocytosis with lymphohistiocytosis
Source: F1000Res. 2024 Apr 23;13:326. [Version 1] doi: 10.12688/f1000research.147189.1 (PMC11487235; doi:10.12688/f1000research.147189.1)
Supplement: Peer review history

# Reviewer report: Reviewer response for version 1

**DOI:** 10.5256/f1000research.161355.r328792

**Published:** 2024-10-17

**Authors:** Ajith Kumar

**Affiliations:** 1 Aster Whitefield Hospital,, Bengaluru, Karnataka, India

It is an interesting case report showing a temporal association of copper sulfate ingestion with the development of secondary HLH. The adapted 2004 HLH guidelines, & H- Score are important tools for helping clinicians arrive at secondary HLH diagnosis. The latter was found to have excellent sensitivity & good specificity in diagnosing secondary HLH. 

 We are not sure if any chemical interaction might have happened between Ethanol and copper sulfate with the resultant compound further causing the observed feature of secondary HLH.

 I would like to know the H-Score calculations, &details about individual components of HScore in this case. Moreover, one needs to be aware that severe sepsis also can cause HLH changes in the bone marrow.

Are enough details provided of any physical examination and diagnostic tests, treatment given and outcomes?

Partly

Is the case presented with sufficient detail to be useful for other practitioners?

Yes

Is sufficient discussion included of the importance of the findings and their relevance to future understanding of disease processes, diagnosis or treatment?

Yes

Is the background of the case’s history and progression described in sufficient detail?

Yes

Reviewer Expertise:

Sepsis/ Shock/ ARDS/ Extra corporeal therapies

I confirm that I have read this submission and believe that I have an appropriate level of expertise to confirm that it is of an acceptable scientific standard.

# Reviewer report: Reviewer response for version 1

**DOI:** 10.5256/f1000research.161355.r288685

**Published:** 2024-06-20

**Authors:** Nur Soyer

**Affiliations:** 1 Ege University, Izmir, Turkey

The history and progression of the case is described in sufficient detail.  Sufficient detail is given about the physical examination and diagnostic tests, the treatment administered and its results.

 There is sufficient discussion of the significance of the findings and their relevance to future understanding of disease processes, diagnosis or treatment. The case is presented in sufficient detail to be useful for other practitioners.

Are enough details provided of any physical examination and diagnostic tests, treatment given and outcomes?

Yes

Is the case presented with sufficient detail to be useful for other practitioners?

Yes

Is sufficient discussion included of the importance of the findings and their relevance to future understanding of disease processes, diagnosis or treatment?

Yes

Is the background of the case’s history and progression described in sufficient detail?

Yes

Reviewer Expertise:

hematological disorders and HLH

I confirm that I have read this submission and believe that I have an appropriate level of expertise to confirm that it is of an acceptable scientific standard.

# Response

**Published:** 2024-06-20

**Authors:** Koushik Ramachandra

**Affiliations:** General Medicine, Kasturba Medical College, Mangalore, Manipal Academy of Higher Education, Manipal, Mangalore, Karnataka, India

Thank you for reviewing our case report Dr. Nur Soyer.
